# Targeting the CD47/SIRPα pathway in malignancies: recent progress, difficulties and future perspectives

**DOI:** 10.3389/fonc.2024.1378647

**Published:** 2024-07-05

**Authors:** Chenyang Jiang, Hao Sun, Zhongxing Jiang, Wenzhi Tian, Shundong Cang, Jifeng Yu

**Affiliations:** ^1^ Department of Hematology, The First Affiliated Hospital of Zhengzhou University, Zhengzhou, China; ^2^ Department of Oncology, Henan Key Laboratory for Precision Medicine in Cancer, Henan Provincial People’s Hospital, Henan University People’s Hospital and Zhengzhou University, Zhengzhou, Henan, China; ^3^ Department of Radiotherapy, The First Affiliated Hospital of Zhengzhou University, Zhengzhou, China; ^4^ ImmuneOnco Biopharmaceuticals (Shanghai) Inc., Shanghai, China

**Keywords:** cd47, SIRPα, signaling pathway, monoclonal antibody, targeted therapy, cancer immunotherapy

## Abstract

Since its initial report in 2015, CD47 has garnered significant attention as an innate immune checkpoint, raising expectations to become the next “PD-1.” The optimistic early stages of clinical development spurred a flurry of licensing deals for CD47-targeted molecules and company mergers or acquisitions for related assets. However, a series of setbacks unfolded recently, starting with the July 2023 announcement of discontinuing the phase 3 ENHANCE study on Magrolimab plus Azacitidine for higher-risk myelodysplastic syndromes (MDS). Subsequently, in August 2023, the termination of the ASPEN-02 program, assessing Evorpacept in combination with Azacitidine in MDS patients, was disclosed due to insufficient improvement compared to Azacitidine alone. These setbacks have cast doubt on the feasibility of targeting CD47 in the industry. In this review, we delve into the challenges of developing CD47-SIRPα-targeted drugs, analyze factors contributing to the mentioned setbacks, discuss future perspectives, and explore potential solutions for enhancing CD47-SIRPα-targeted drug development.

## 1 Introduction

Immunotherapy has emerged as the primary treatment approach for various cancers, significantly altering survival outcomes for numerous cancer types. A rising number of patients now experience prolonged survival without active therapy, hinting at the potential for curing even stage IV diseases. However, the majority still succumbs to cancer, primarily due to the limitations of current immune checkpoint inhibitors (ICIs) immunotherapies (1, 2). Recent findings underscore the pivotal role of CD47 in innate and adaptive immune escape, functioning as an immunological checkpoint (3). Conversely, signal regulatory protein α (SIRPα) is an Ig-like protein highly expressed in macrophages, dendritic cells, neutrophils, and neurons (4–6). The CD47/SIRPα signaling pathway through CD47 interacting with SIRPα on macrophages and dendritic cells, is an attractive target for cancer therapies in both solid tumors and hematological malignancies by regulating normal immune tolerance and autoimmunity (4, 7–10). Numerous studies of CD47/SIRPα blockades by targeting either CD47 or SIRPα have demonstrated potent anti-tumor action in preclinical models and clinical studies (4, 11, 12). However, numerous setbacks have been reported regarding this targeted therapy which not only raised doubts in the industry about CD47 targeted drug development plan but also various interpretations pointing to the druggability of CD47 targets. In this review, we highlight the recent progress and difficulties in CD47/SIRPα targeted drug development for cancer immunotherapy, look into future perspectives, and explore potential solutions to improve CD47/SIRPα targeted drug development.

## 2 Targeting CD47/SIRPα signaling pathway in cancer immunotherapy

“Don’t eat me” signaling pathways refer to mechanisms employed by cancer cells to evade detection and destruction by the immune system. Some key pathways have been identified and explored for their implications in immunotherapy, including the CD47-SIRPα axis, the Programmed cell death ligand 1 (PD-L1)/programmed cell death protein 1 (PD-1) pathway, the CD24/Siglec axis, and others. Each of these signaling pathways offers advantages in cancer immunotherapy, and a list of agents under development for these pathways is summarized in **Table 1**. In summary, targeting “don’t eat me” signaling pathways in cancer immunotherapy holds promise for enhancing anti-tumor immune responses. However, the efficacy of these approaches may vary among different cancer types and individuals, and further research is needed to optimize their therapeutic potential and minimize potential side effects.

**Table 1:** List of “don’t eat me signaling pathways.

Pathway	Description	Pros in Immunotherapy	Cons in Immunotherapy	Example agents under development
CD47-SIRPα	CD47 is a cell surface protein expressed on cancer cells that interacts with SIRPα on macrophages, delivering a “don’t eat me” signal and inhibiting phagocytosis.	Targeting CD47 with monoclonal antibodies or other inhibitors can enhance macrophage-mediated phagocytosis of cancer cells, promoting tumor clearance.	Some studies suggest that blocking CD47-SIRPα interaction may lead to off-target effects and autoimmune reactions if normal cells are inadvertently targeted. Additionally, cancer cells may develop resistance mechanisms, such as upregulation of alternative “don’t eat me” signals.	CD47 targeting agents: Magrolimab (Hu5F9-G4), ALX148, TTI-621 (SIRPαFc), TTI-622 (SIRPα-IgG4 Fc), CC-90002, SRF231, PF-06650808 (TTI-621). SIRPα targeting agents: ALX148, BI 765063, IMM01
PD-L1/PD-1	PD-L1 expressed on cancer cells binds to PD-1 on T cells, leading to T cell exhaustion and immune evasion.	Immune checkpoint inhibitors targeting PD-1 or PD-L1 can block this interaction, reactivating T cells and enhancing anti-tumor immune responses.	Response rates to PD-1/PD-L1 inhibitors vary among different cancer types, and not all patients respond to treatment. Furthermore, some tumors may utilize alternative immune evasion mechanisms, limiting the efficacy of PD-1/PD-L1 blockade alone.	PD-1 Inhibitors: Pembrolizumab (Keytruda), Nivolumab (Opdivo), Cemiplimab (Libtayo), Sintilimab, Toripalimab and Camrelizumab. PD-L1 Inhibitors: Atezolizumab (Tecentriq), Durvalumab (Imfinzi), Avelumab (Bavencio), Envafolimab, KN035 (Envafolimab), and BGB-A333.
CD24-Siglec	CD24, a glycoprotein expressed on cancer cells, interacts with Siglec receptors on immune cells, inhibiting their activation and promoting immune evasion.	Blocking CD24-Siglec interactions may enhance anti-tumor immune responses, similar to strategies targeting CD47 or PD-L1/PD-1.	This pathway is relatively less studied compared to CD47-SIRPα or PD-L1/PD-1, and further research is needed to fully understand its therapeutic potential and potential side effects.	CD24Fc (STC009), ALM201, Bi-specific antibodies, antibody-drug conjugates.
HLA-G	Cancer cells may upregulate expression of the human leukocyte antigen-G (HLA-G), which interacts with inhibitory receptors on immune cells, such as ILT2 and KIR2DL4, to suppress immune responses.	Targeting HLA-G or its receptors may enhance anti-tumor immune responses.	Similar to other “don’t eat me” pathways, off-target effects and the potential for autoimmune reactions are concerns.	Kusacitinib (ASP1235/AGS-16C3F), IER3IP1, Monalizumab, anti-HLA-G antibodies

**Figure 1:**
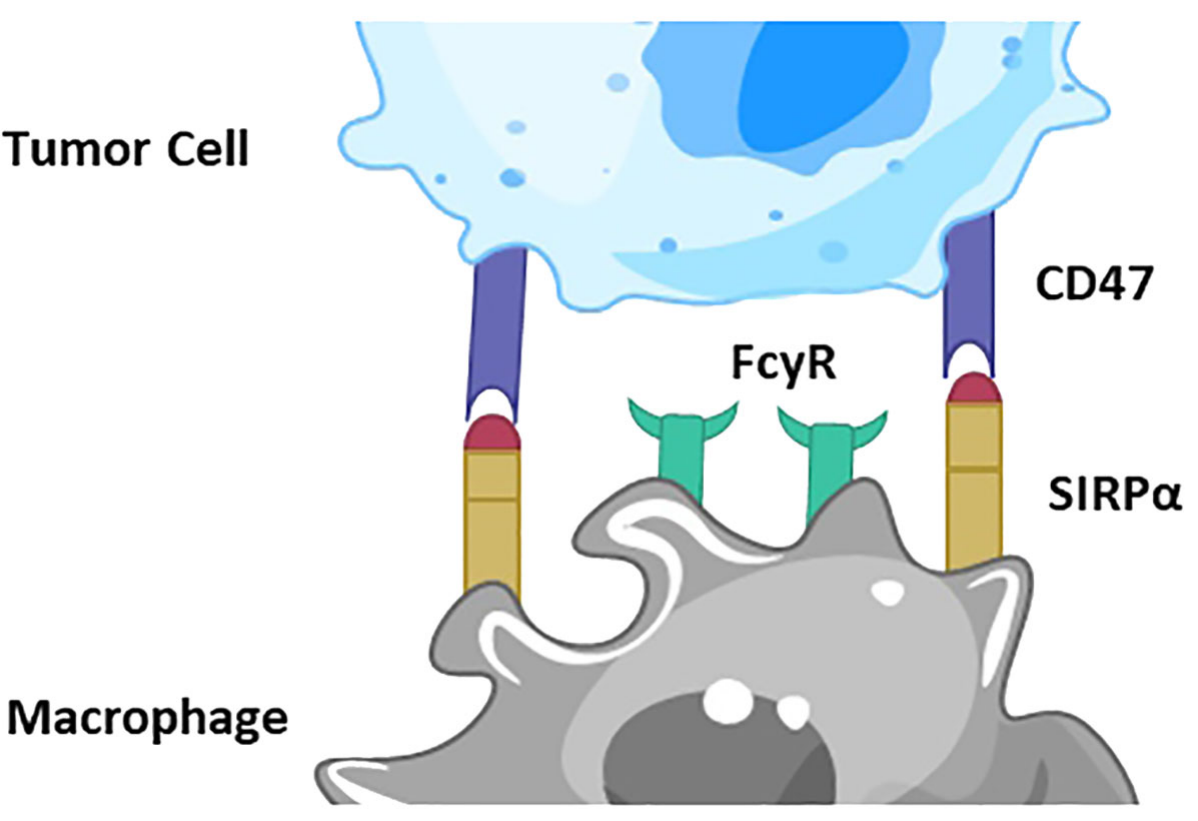
Depiction of CD47/SIRPα signaling pathway. CD47 is overexpressed on the surface of cancer cells, acting as a “don’t eat me” signal. SIRPα is a receptor on the surface of macrophages and dendritic cells. When CD47 binds to SIRPα, it sends an inhibitory signal to the myeloid cells. This interaction inhibits the phagocytic activity of the myeloid cells, preventing them from engulfing and destroying cancer cells, thus allowing the cancer cells to evade the immune system.

The CD47/SIRPα pathway plays a crucial role in regulating immune responses, particularly in the context of cancer immunotherapy. Briefly, CD47 is often overexpressed on the surface of cancer cells, acting as a “don’t eat me” signal. SIRPα is a receptor found on the surface of myeloid cells, such as macrophages and dendritic cells. When CD47 binds to SIRPα, it sends an inhibitory signal to the myeloid cells. This interaction inhibits the phagocytic activity of the myeloid cells, preventing them from engulfing and destroying cancer cells, thus allowing the cancer cells to evade the immune system (**Figure 1**).

In immunotherapy, blocking the CD47/SIRPα interaction can enhance the immune system’s ability to target and eliminate cancer cells. Anti-CD47 antibodies or SIRPα-Fc fusion proteins can be used to block this pathway. By inhibiting the CD47/SIRPα interaction, the “don’t eat me” signal is turned off, allowing macrophages and other myeloid cells to recognize and phagocytose cancer cells more effectively. This mechanism makes the CD47/SIRPα pathway a promising target for cancer immunotherapies, aiming to boost the natural immune response against tumors.

As a potent “don’t eat me” anti-phagocytosis messaging signal, CD47 serves its functions by interacting with SIRPα on macrophages and dendritic cells. This signaling pathway is an attractive target for cancer therapies as it serves roles in both solid tumors and hematological malignancies in addition to its involvement in regulating normal immune tolerance and autoimmunity (4, 5, 13–15).

**Figure 2:**
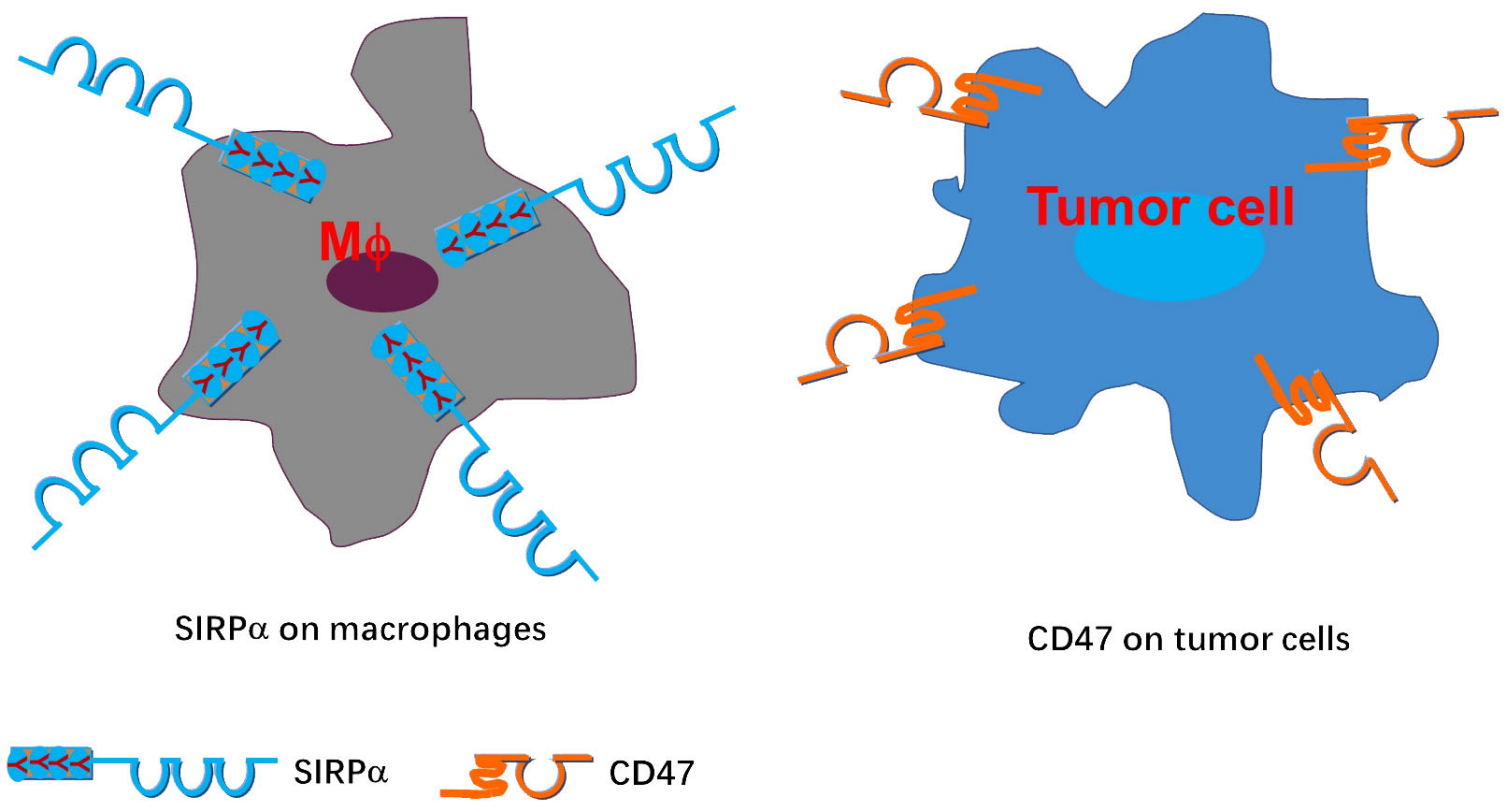
Depiction of CD47 and SIRPα structures.

The structures of CD47 and signal regulatory protein alpha (SIRPα) have been extensively reported in various publications (5–9, 16). CD47, also known as integrin-associated protein (IAP), is an Ig-like protein with widespread expression, including T cells, B cells, red blood cells, platelets, and hematopoietic stem cells. Conversely, SIRPα is an Ig-like protein highly expressed in macrophages, dendritic cells, neutrophils, and neurons (**Figure 2**) (7, 10, 16). Recent advancements, such as the crystallization of CD47-targeting agent IMM01, provide a structural basis for potential clinical applications (16).

**Figure 3:**
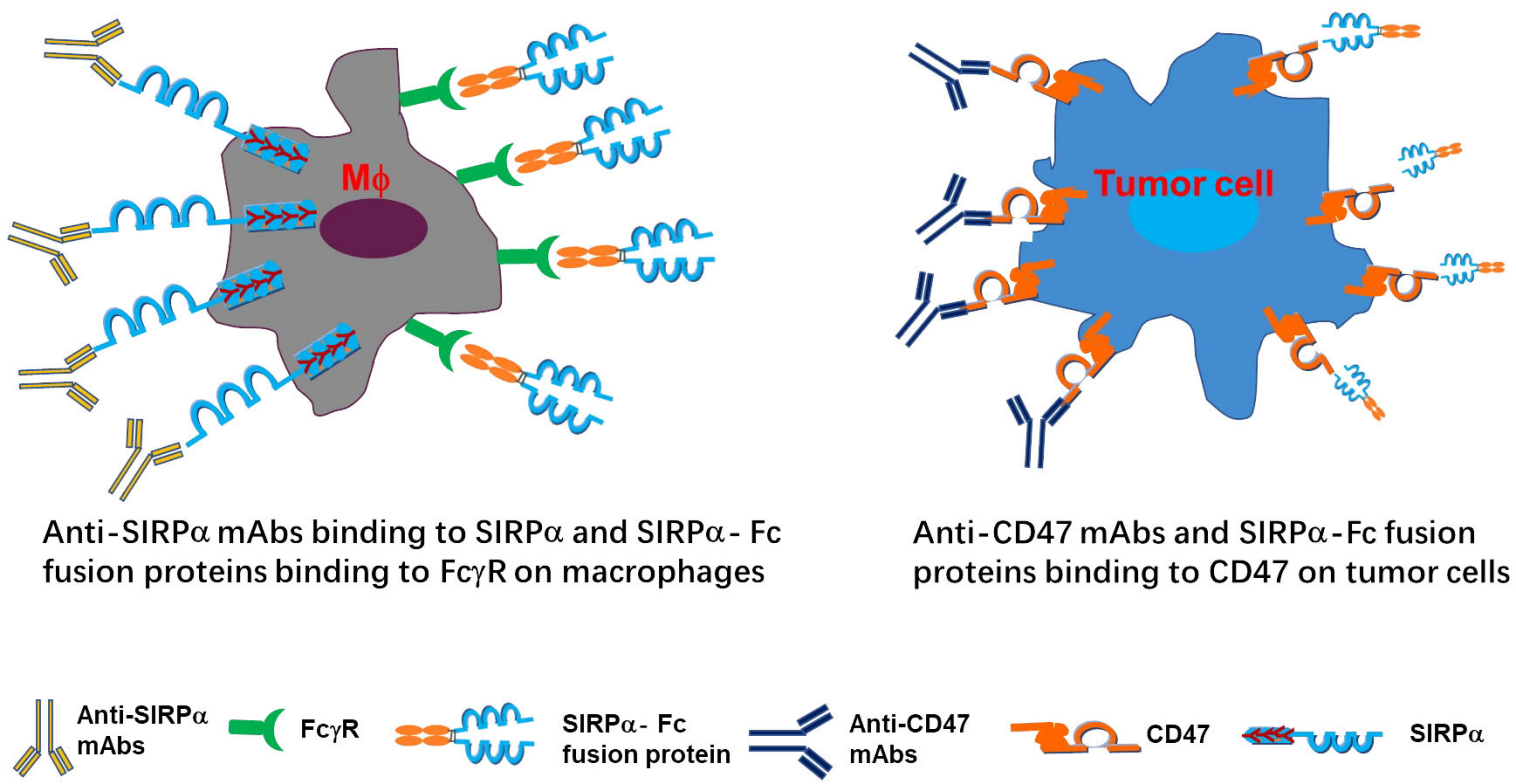
Depiction of mechanism of actions targeting CD47/SIRPα axis. The anti-SIRPα mAbs bind to SIRPα and SIRPα-Fc fusion proteins bind to FcγR on macrophages (left panel), while anti-CD47 mAbs SIRPα-Fc fusion proteins bind to CD47 on tumor cells (right panel).

The CD47/SIRPα signaling pathway plays a crucial role in regulating myeloid cell-target cell signal transduction, as depicted in **Figure 3** (7, 14). This axis has broad implications in the maintenance of erythrocytes, platelets, and hematopoietic stem cells. The expression of CD47 on cancer cells, akin to the PD-1/PD-L1 interaction inhibiting T cell activity, can impede myeloid cell-mediated clearance. Numerous studies have delved into the mechanisms of action of CD47/SIRPα blockades by targeting either CD47 or SIRPα. Various promising molecules blocking the CD47-SIRPα axis are in pre-clinical development.

Monoclonal antibodies and fusion proteins blocking CD47/SIRPα signaling have demonstrated potent anti-tumor action in preclinical models and clinical studies (7, 11, 12). Clinical trials have been initiated to investigate the effects of combining Azacitidine (Aza), an anti-metabolite chemotherapy drug, with CD47/SIRPα inhibition, such as the anti-CD47 monoclonal antibody (mAb) Magrolimab, in patients with hematologic malignancies. Although anemia was a frequent side effect of anti-CD47 therapy, risk-mitigation strategies such as using a low “priming” dose have significantly decreased this risk in clinical studies (17).

However, numerous setbacks have been encountered and reported regarding this targeted therapy which not only raised doubts in the industry about CD47 targeted drug development plan but also various interpretations pointing to the druggability of CD47 targets. With that, first, the phase II trial plan for CD47 mAb ZL-1201 was cancelled in August 2022 (18); then, in July 2022, the developer of ZL-1201 decided to deprioritize ZL-1201 for internal development but would pursue out-licensing opportunities; furthermore, in January 2023, another developer abandoned the clinical development of CD47 antibodies and dismissed all employees. Intense debates in the industry were further triggered by the termination of the phase III clinical study ENHANCE in which the combination of Magrolimab and Aza was tested against Aza alone as the first-line treatment for high-risk myelodysplastic syndrome (MDS) (19). How can we approach the viability of CD47 targets with greater objectivity and reasoning? In this review, we highlight the difficulties in CD47/SIRPα targeted drug development for cancer immunotherapy, look into the future perspectives and explore potential solutions to improve CD47-SIRPα targeted drug development.

## 3 Clinical development of CD47/SIRPα targeted agents

The landscape of clinical development for CD47/SIRPα targeted agents has witnessed a substantial surge in recent years, with numerous candidates entering clinical trials. The clinicaltrial.gov registry currently lists 38 ongoing trials targeting CD47 and 6 trials targeting SIRPα. This new generation of CD47 antagonist products falls into five categories (12, 20) (**Table 2**):

**Table 2:** Categories for CD47 antagonist products.

Categories	CD47 targeted agents
CD47 mAb	CC-90002 (INBRX-103), GenSci-059, IMC-002 (3D-197), Lemzoparlimab (TJ011133; TJC4), Letaplimab (IBI-188), Ligufalimab (AK117), Magrolimab (Hu5F9-G4), MIL-95 (CM-312), SHR-1603 and ZL-1201.
CD47-targeted BsAbs	BAT-7104 (BAT-7101), HX-009 (HX-009–5), IBI-322, IMM-0306, JMT-601 (CPO-107), SG-12473, TG-1801 (NI-1701), XL-114 (AU-341, AU7R-104 and AUR-104).
SIRPα/Fc fusion protein antibody	Evorpacept (ALX-148), IMM-01, TTI-621 and TTI-622.
CD47 small-molecule inhibitor	RRx-001
CD47 ADCs	anti-CD47 mAb-DM1

mAb, monoclonal antibody; BsAbs, bispecific antibodies; ADCs, antibody-drug conjugates.

As of October 2023, there are no publicly published clinical trials on CD47 small-molecule inhibitors or ADC specifically for hematologic malignancies. Notably, SIRP/Fc fusion protein antibodies possess the capability to block CD47/SIRPα binding, thereby reducing the “don’t eat me” signal from CD47 and activating a pro-phagocytic signal via Fc receptors (7, 21). These antibodies exhibit low binding to red blood cells (RBCs) or blood platelets, distinguishing them from anti-CD47 monoclonal antibodies due to the absence of SIRPα expression on human blood cells (22).

Several anti-CD47 mAb products are actively undergoing clinical development in various forms, excluding acute myeloid leukemia (AML). Examples include AO-176 (CD47 mAb) (23), TG1801 (anti-CD19xCD47 bispecific), IMC-002 (CD47 mAb), STI-6643 (CD47 mAb), SL-172154 (SIRPα-Fc-CD40L), ZL-1201 (CD47 mAb), IMM01 (SIRPα-Fc fusion protein), PF-07257876 with IBI322 (anti-CD47 and PD-L1), and IMM-0306 (CD47xCD20 bispecific). Additionally, products targeting SIRPα, such as GS-0189, CC-95251, and BI765063/OSE-172, are also actively undergoing clinical development.

## 4 CD47-SIRPα targeted immunotherapy in solid tumors

### 4.1 CD47 targeted therapy in ovarian cancer

CD47 has been identified as a specific and overexpressed marker on ovarian cancer (OC) cells since 1986 (24), with factors such as Myc, NF-B, and HIF-1 regulating its expression (25–29). In the realm of CD47 targeted therapy for ovarian cancer, various approaches have been explored, ranging from monotherapy inhibiting the CD47-SIRPα axis to combinations with tumor-targeting antibodies and bispecific antibodies disrupting the CD47-SIRPα axis (30). As of August 19, 2023, four CD47 targeted treatment trials for ovarian cancer were registered in the US National Clinical Trials Registry (NCT), including BI 765063 (an antibody antagonist of SIRPα formerly known as OSE-172, NCT04653142), which exhibited potential effectiveness and good tolerance in its preliminary report without dose-limiting toxicities in patients with ovarian cancer (**Table 3**) (31). Notably, BI 765063 is also undergoing clinical trials for other cancer types (NCT03990233, NCT05249426, NCT05446129, and NCT05068102).

**Table 3:** CD47 antagonists currently entering clinical trials for treatment of ovarian cancers.

NCT (CTR)	Drug Name (Alternative name)	Phase	Study Status	Enrollment	Companies	Target-based Actions	Drug Classification	Indications
NCT05403554	NI-1801	Phase I	Recruiting	40	Light Chain Bioscience - Novimmune SA	CD47 antagonist	anti-CD47/MSLN BsAb	OC/NSCLC
NCT04881045	PF-07257876	Phase I	Active_not_recruiting	28	Pfizer	CD47 antagonist	anti-CD47/PD-L1 BsAb	OC/NSCLC
NCT05261490	TTI-622 (PF-07901801)	Phase I/II	Active_not_recruiting	50	Pfizer	CD47 antagonist	anti-CD47 mAb	OC
NCT05467670	ALX148	Phase II	Recruiting	31	ALX Oncology	SIRPα-Fc fusion protein,SIRPα/CD47 blocker	SIRPα/Fc fusion protein antibody	OC

MSLN, Mesothelin; PD-L1, Programmed death-ligand 1; NSCLC, Non-Small Cell Lung Cancer; OC, Ovarian Cancer; BsAb, Bispecific Antibody; mAb, monoclonal antibody.

Monotherapy involving the blockade of the CD47-SIRPα axis has shown promising outcomes in ovarian cancer, with the combination of pro-phagocytic CD47-SIRPα axis monoclonal antibodies and Fc-dependent effects of tumor-targeting antibodies, such as antibody-dependent cellular cytotoxicity (ADCC) and antibody-dependent cellular phagocytosis (ADCP), demonstrating synergistic efficacy (10, 24). In a phase Ib trial involving 18 platinum-resistant or refractory OC patients, the combination of Hu5F9-G4 with Avelumab (PD-L1) showcased a 56% stable disease (SD) rate (32), prompting further evaluation in a phase II trial (NCT03558139). Another promising candidate, PF-07257876, a dual checkpoint inhibitory bispecific antibody simultaneously blocking CD47 and PD-L1, is currently undergoing a phase I dose escalation and expansion trial in OC patients (NCT04881045).

### 4.2 Targeting CD47 in breast cancer treatments

In the realm of breast cancer treatments, a preclinical study suggests that overcoming resistance to trastuzumab in HER2/Neu+ breast cancer can be achieved by combining CD47 blockade with trastuzumab (33). The combination of CD47 mAb with trastuzumab not only significantly reduced the growth of HER2^+^ breast tumors resistant to ADCC but also enhanced the efficacy of trastuzumab, leading to complete tumor regression (33, 34).

In the case of triple-negative breast cancer (TNBC), cells that had previously undergone chemotherapy showed increased CD47 expression (35, 36). Preclinical studies have demonstrated that anti-CD47 mAb decreases breast cancer cell proliferation and induces the death of breast cancer cells *in vitro* (37). The synergistic effect of anthracyclines and CD47 mAb has been observed to enhance tumor ablation *in vivo* (38, 39). In other preclinical studies, the combination of CD47 mAb and cabazitaxel exhibited a significant anti-cancer effect in TNBC (40). Antibody-drug conjugates (ADCs) utilizing mertansine and anti-CD47 mAb for TNBC treatment demonstrated potent tumor suppressive activity without systemic immune toxicity (20). Furthermore, *in vivo* studies have shown that inhibiting CD47 successfully reverses radiation resistance and improves phagocytosis (41). Currently, five products targeting the CD47-SIRPα signaling pathway are undergoing various clinical trials for breast cancer treatment (**Table 4**). These trials reflect the growing interest and exploration of CD47-SIRPα targeted immunotherapy in the context of breast cancer. In addition, CD47 inhibitors are also under evaluation on multiple other malignancies, such as head and neck squamous cell carcinoma.

**Table 4:** CD47 related ongoing clinical trials in breast cancers.

NCT	Drug Name (Alternative name)	Phase	Study Status	Enrollment	Companies	Target-based Actions	Drug Classification	Indications
NCT05765851	DS-1103a	Phase I	Recruiting	78	Daiichi Sankyo, Inc.	CD47/SIRPa antagonist	anti-SIRPα mAb	BC, Advanced Solid Tumor
NCT05403554	NI-1801	Phase I	Recruiting	40	Light Chain Bioscience - Novimmune SA	CD47 antagonist	anti-CD47/MSLN BsAb	Epithelial OC, TNBC, NSCLC
NCT05780307	IMM2520	Phase I	Recruiting	48	ImmuneOnco Biopharmaceuticals (Shanghai) Inc.	CD47/SIRPa antagonist	anti-CD47/PD-L1 BsAb	Advanced Solid Tumor, NSCLC, BC, HNSCC, CRC
NCT04980690	IBC0966	Phase I/II	Recruiting	228	SunHo BioPharma BioPharmaceutical CO., Ltd.	CD47/SIRPa antagonist	anti-CD47/PD-L1 BsAb	Advanced Malignant Tumors, including TNBC
NCT05807126	Magrolimab (Hu5F9-G4)	Phase I	Recruiting	33	Gilead Sciences	CD47 antagonist	anti-CD47 mAb	Stage IV BC

OC, Ovarian Cancer; TNBC, Triple Negative Breast Cancer; HNSCC, Head and Neck Squamous Cell Carcinoma; CRC, Colorectal Cancer; NSCLC, Non-small Cell Lung Cancer; MSLN, mesothelin; BsAb, Bispecific Antibody; mAb, monoclonal antibody.

## 5 Targeting CD47/SIRPα immunotherapy in hematological malignancies

### 5.1 Targeting CD47 immunotherapy in lymphoma

In the realm of targeting CD47 immunotherapy for lymphomas, there are currently 15 CD47 antagonists undergoing clinical studies for treatment (**Table 5**). Some CD47 monoclonal antibodies, including CC-90002, Hu5F9-G4 (Magrolimab), TJ011133 (Lemzoparlimab) (33). and AK117 (Ligufalimab), have recently initiated clinical trials (12).

**Table 5:** CD47 antagonists currently entering clinical trials for treatment of lymphomas.

NCT	Drug Name (Alternative name)	Phase	Study Status	Enrollment	Companies	Target-based Actions	Drug Classification	Indications
NCT03013218	Evorpacept (ALX-148)	Phase I	Active_not_recruiting	174	ALX Oncology	SIRPα-Fc fusion protein,SIRPα/CD47 blocker	SIRPα/Fc fusion protein antibody	NHL
NCT05025800	Evorpacept (ALX-148)	Phase I/II	Recruiting	47	ALX Oncology	SIRPα-Fc fusion protein,SIRPα/CD47 blocker	SIRPα/Fc fusion protein antibody	B-Cell NHL
NCT05221385	GenSci-059	Phase I	Recruiting	58	GeneScience Pharmaceuticals Co Ltd	CD47 antagonist	anti-CD47 mAb	Lymphoma
NCT05189093	HX-009 (HX-009–5)	Phase II	Recruiting	99	HanX Biopharmaceuticals Inc	CD47 antagonist	anti-CD47/PD-L1 BsAb	Lymphoma
NCT04306224	IMC-002 (3D-197)	Phase I	UNKNOWN	24	ImmuneOncia Therapeutics LLC	CD47 antagonist	anti-CD47 mAb	Lymphoma
NCT05833984	IMM-01	Phase I/II	Recruiting	309	ImmuneOnco Biopharm Co Ltd	CD47 antagonist	SIRPα/Fc fusion protein antibody	HL
NCT04746131	IMM-0306	Phase I	IMM-0306		ImmuneOnco Biopharm Co Ltd	CD47 antagonist	anti-CD47/CD20 BsAb	CD20-positive B-cell NHL
NCT04853329	JMT-601 (CPO-107)	Phase II	CPO-107		Shanghai JMT-Bio Inc	CD47 antagonist	anti-CD47/CD20 BsAb	CD20 positive B-cell NHL
NCT04728334	Ligufalimab (AK117)	Phase I	Active_not_recruiting	49	Akeso	CD47 antagonist	anti-CD47 mAb	NHL
NCT04788043	Magrolimab (Hu5F9-G4)	Phase II	Recruiting	24	Gilead Sciences	CD47 antagonist	anti-CD47 mAb	HL
NCT02953509	Magrolimab (Hu5F9-G4)	Phase I/II	Active_not_recruiting	178	Gilead Sciences	CD47 antagonist	anti-CD47 mAb	NHL
NCT04651348	MIL-95 (CM-312)	Phase I	Recruiting	58	KeyMed Biosciences Co Ltd	CD47 antagonist	anti-CD47 mAb	Lymphoma
NCT03804996	TG-1801 (NI-1701)	Phase I	Active_not_recruiting	50	TG Therapeutics Inc	CD47 antagonist	anti-CD47/CD19 BsAb	B-Cell Lymphoma
NCT04806035	TG-1801 (NI-1701)	Phase I	Recruiting	60	TG Therapeutics Inc	CD47 antagonist	anti-CD47/CD19 BsAb	B-Cell Lymphoma
NCT03530683	TTI-622	Phase I	Active_not_recruiting	177	Trillium Therapeutics Inc	CD47 antagonist	SIRPα/Fc fusion protein antibody	Lymphoma

HL, Hodgkin Lymphoma; NHL, non-Hodgkin Lymphoma; mAb, monoclonal antibody; BsAb, Bispecific antibody.

Magrolimab (Hu5F9-G4), a humanized-IgG4 antibody, has shown promising activity in both aggressive and indolent non-Hodgkin lymphoma (NHL) patients in a phase Ib trial (NCT02953509) (17, 42). The combination of Magrolimab with rituximab demonstrated an overall response rate (ORR) of 50% and a complete response rate (CR) of 36% in patients with relapsed/refractory (R/R) lymphoma. Notably, patients with diffuse large B-cell lymphoma (DLBCL) had an ORR of 40% and a CR of 33%, while patients with follicular lymphoma (FL) exhibited an ORR of 71% and a CR of 43% (15, 17, 43). Additionally, Bispecific antibodies (BsAbs) simultaneously blocking the CD47-SIRPα axis and binding to the tumor-specific antigen (CD20) have shown promising efficacy with less blood cell destruction in a human NHL-engrafted mouse model (44).

CC-90002, a CD47 monoclonal antibody, in combination with rituximab (CD20 monoclonal antibody), has been studied in a phase I trial for patients with heavily pretreated R/R NHL, showing good tolerance and modest clinical activity (45). TJ011133 (Lemzoparlimab) has been examined in a phase Ib clinical trial (NCT03934814) for R/R patients with CD20-positive NHL, demonstrating therapeutic efficacy, good tolerance, and controllable side effects, with an ORR of 57% (46). These ongoing clinical trials underscore the growing interest and potential of CD47-SIRPα targeted immunotherapy in the treatment of lymphomas, presenting new avenues for improving patient outcomes.

AK117, currently in a phase I clinical trial, has demonstrated good tolerance with no hematological adverse events (AEs). Being tested for various hematologic malignancies, AK117 is undergoing clinical trials either as a monotherapy or in combination with other agents, such as rituximab (47).

IMM0306, a fusion protein of CD20 monoclonal antibody with the CD47 binding domain of SIRPα, demonstrated excellent cancer-killing efficacy by activating both macrophages and NK cells (48). Clinical trials (NCT05771883 and NCT05805943) are underway to evaluate the safety and efficacy of IMM0306 in combination with lenalidomide and as a monotherapy in patients with R/R CD20-positive B-cell NHL, respectively. Clinical trials (NCT05771883 and NCT05805943) are underway to evaluate the safety and efficacy of IMM0306 in combination with lenalidomide and as a monotherapy in patients with R/R CD20-positive B-cell NHL, respectively (1). IMM0306 has displayed superior tumor cell binding preference and more effective anti-lymphoma activity compared to CD47 monoclonal antibody alone in both *in vivo* and *in vitro* studies (44, 49). IMM0306 activates macrophages and NK cells by blocking the connection between CD47 and SIRPα and engaging FcγR, resulting in excellent cancer-killing effectiveness without binding activity on human RBCs and no hemolytic side effects. When compared to rituximab, IMM0306 shown stronger ADCC activity and reduced CDC activity in several hematologic malignancy cells, presumably, because the IMM0306’s Fc portion is an IgG1 molecule. In combination with lenalidomide, IMM0306 has shown greater efficacy than any single treatment or rituximab in combination with lenalidomide in an orthotopic lymphoma model (9). Phase I clinical trials for IMM0306 have been initiated in the United States and China for patients with R/R CD20-positive B-cell NHL (NCT05771883, NCT05805943, and CTR20192612).

The anti-CD47/CD19 BsAb TG-1801 (NI-1701) has demonstrated strong potential in preclinical studies to elicit ADCP and ADCC in malignant B-cell lines and primary tumor B-cells from patients with acute lymphoblastic leukemia (ALL), chronic lymphocytic leukemia (CLL), and several subtypes of NHL (7, 50, 51). Two phase I trials (NCT03804996 and NCT04806035) are currently evaluating TG-1801’s safety and effectiveness in treating patients with B-cell lymphoma and CLL.

Four SIRPα/Fc fusion protein antibodies, TTI-621, TTI-622, ALX418, and IMM01, are undergoing clinical testing. These fusion proteins, created by combining the CD47-binding domain of human SIRPα with either the human IgG1 or IgG4 Fc region, aim to enhance phagocytosis and anti-cancer activity of macrophages by blocking CD47-SIRPα contact between malignant cells and macrophages through Fc receptor engagement (9, 21, 52–55). In a phase I study of TTI-621 in patients with R/R lymphoma, favorable safety and efficacy profiles were observed, with 14 out of 71 NHL patients showing responses and a 20% ORR (21, 56).

TTI-622 is currently undergoing a multicenter phase I dose-escalation and expansion trial (NCT03530683). According to preliminary findings, out of the nine patients, two achieved a CR (one in DLBCL and one in cutaneous T-cell lymphoma—mycosis fungoides), and seven achieved a partial response (PR) (two each in cutaneous T-cell lymphoma, peripheral T-cell lymphoma, DLBCL, and FL) (53).

ALX148, also known as Evorpacept, is a CD47-blocking molecule with a modified SIRP D1 domain connecting to an inactive human IgG1 Fc. In a phase I clinical trial (NCT03013218) involving 33 patients receiving various doses of ALX148 along with rituximab, it demonstrated promising anti-tumor efficacy and low adverse events (57).

IMM01, a recombinant human SIRP-IgG1 fusion protein, has shown potent dual-functional anti-tumor action by inducing phagocytosis in preclinical experiments. It also exhibited the ability to weakly bind human erythrocytes without significant hemolysis (9, 16). According to preliminary findings from a phase I study (CTR1900024904) involving 14 patients with R/R lymphoma, the ORR was 14.3% (one CR and one PR), with two patients at validated SD (58). Additionally, preliminary data from a phase II clinical trial with IMM01 demonstrated a robust anti-tumor effectiveness with a well-tolerated safety profile in classical Hodgkin lymphoma patients who had failed prior anti-PD-1 therapy (59).

### 5.2 Targeting CD47/SIRP *α* immunotherapy in leukemia and MDS

CD47 is highly expressed on AML cells, including leukemic stem cells, in 25–30% of AML patients, serving as an independent prognostic factor for poor overall survival (60). Currently, 11 CD47 antagonists are undergoing clinical trials for the treatment of hematological malignancies (**Table 6**), with published clinical research data available for some of these CD47 antagonists (7, 9, 12, 17, 43, 61).

**Table 6:** CD47 agents currently entering clinical trials for treatment of hematological malignancies except lymphoma.

NCT	Drug Name (Alternative name)	Phase	Study Status	Enrollment	Companies	Target-based Actions	Drug Classification	Indications
NCT05607199	AUR103	Phase I	Recruiting	80	Aurigene Discovery Technologies Limited	CD47 antagonist	Small molecule CD47 antagonist	AML/MDS
NCT05266274	CD47 mAb	NA	Recruiting	69	The First Affiliated Hospital of Soochow University	CD47 antagonist	anti-CD47 monoclonal antibody	AML after transplantation
NCT04417517	Evorpacept (ALX-148)	Phase I/II	Active_not_recruiting	65	ALX Oncology Inc.	SIRPα-Fc fusion protein,SIRPα/CD47 blocker	SIRPα/Fc fusion protein antibody	Higher Risk MDS
NCT04755244	Evorpacept (ALX-148)	Phase I/II	Active_not_recruiting	97	ALX Oncology Inc.	SIRPα-Fc fusion protein,SIRPα/CD47 blocker	SIRPα/Fc fusion protein antibody	AML
NCT04900350	Ligufalimab (AK117)	Phase I/II	Recruiting	190	Akeso	CD47 antagonist	anti-CD47 monoclonal antibody	MDS
NCT04980885	Ligufalimab (AK117)	Phase I/II	Recruiting	160	Akeso	CD47 antagonist	anti-CD47 monoclonal antibody	AML
NCT05367401	Magrolimab (Hu5F9-G4)	Phase I/II	Not_yet_recruiting	63	Gilead Sciences	CD47 antagonist	anti-CD47 monoclonal antibody	MDS/AML
NCT05823480	Magrolimab (Hu5F9-G4)	PHASE1	Not_yet_recruiting	44	Gilead Sciences	CD47 antagonist	anti-CD47 monoclonal antibody	AML/MDS
NCT04599634	Magrolimab (Hu5F9-G4)	Phase I	Recruiting	76	Gilead Sciences	CD47 antagonist	anti-CD47 monoclonal antibody	CLL/B-Cell Lymphoma
NCT05139225	TTI-622	Phase I	Recruiting	32	Trillium Therapeutics Inc	CD47 antagonist; Immunoglobulin gamma Fc receptor agonist	CD47 antagonist; Immunoglobulin gamma Fc receptor agonist	MM
NCT05675449	TTI-622	Phase I	Recruiting	14	Trillium Therapeutics Inc	CD47 antagonist; Immunoglobulin gamma Fc receptor agonist	CD47 antagonist; Immunoglobulin gamma Fc receptor agonist	MM

AML, Acute Myeloid Leukemia; MDS, Myelodysplastic Syndromes;MM, Multiple Myeloma; CLL, Chronic Lymphocytic Lymphoma; mAb, Monoclonal Antibody.

Magrolimab (5F9), a humanized IgG4 anti-CD47 antibody, demonstrated leukemic eradication and long-term survival in a preclinical xenografted mouse model (62). In a phase I monotherapy trial for patients with R/R AML, Magrolimab did not achieve CR (63). However, promising ORR were observed in combination with Aza for untreated AML in phase Ib trials (64). The ORR was 65% among 34 evaluable patients, with a higher CR rate in TP53-mutated AML patients. Subsequent trials showed 53% and 56% CR or incomplete count recovery (CRi) (63). Subsequent trials showed 53% and 56% CR or CRi rates in AML/MDS patients, with higher rates in those with TP53 mutations (63). In another study with 15 patients enrolled, the CR/CRi rate in the newly diagnosed AML patients was 100% (15/15) with a CR rate of 87% (13/15). Patients with TP53 mutations achieved a CR/CRi of 100% (7/7) and a CR of 86% (6/7) (65). In R/R prior venetoclax (VEN) failure AML, the CR/CRi rate was 27% (3/13) with a median overall survival (OS) of 3.1 months (range 0.9–6.5) (66). Magrolimab has progressed to phase III trials.

CD47 mAb IBI-188 (Letaplimab) is undergoing clinical trials for newly diagnosed intermediate and high-risk MDS and AML. Phase III trials in China for hematologic disorders and phase I trials with rituximab in advanced lymphoma, Aza in AML, and Aza in newly diagnosed higher-risk MDS are also ongoing (NCT03717103, NCT04485052 and NCT04485065).

CD47 mAb TJ011133 (TJC4, Lemzoparlimab) is being tested in a phase I trial for R/R AML or MDS, showing promising efficacy and manageable adverse events (NCT04202003). Four of five patients had treatment-related AEs with one AE of grade 3 while the rest at grades 1–2. One R/R AML patient attained morphologic leukemia-free status following two cycles of therapy (67). A phase III trial in primary higher-risk MDS is planned.

CC-90002, a newer CD47 antagonist, demonstrated potential efficacy in preclinical models but a phase I study for R/R AML and MDS was terminated due to lack of monotherapy activity and evidence of anti-drug antibodies (68–70). At present, no clinical trial of CC-90002 was found in hematological malignancies.

In a phase Ib trial, anti-CD47 mAb AK117 in combination with Aza demonstrated favorable tolerance and a 54.2% CR rate in newly diagnosed high-risk MDS patients (71).

Targeting the CD47-SIRPα pathway with recombinant fusion proteins (e.g., TTI-662)) and bispecific antibodies has entered clinical trials (72, 73). Preliminary data for the SIRPα-Fc fusion protein IMM01 in combination with Aza showed promising efficacy and tolerability in patients with treatment-naive chronic myelomonocytic leukemia (CMML) and higher-risk myelodysplastic syndrome (HR-MDS). Among the 16 efficacy-evaluable CMML patients with treatment duration ≥3 months, the ORR was 87.5%, and the CR rate reached 25.0% (74). In another study IMM01 (without low-dose priming) in combination with Aza showcased promising efficacy in patients with treatment-naive HR-MDS. Among the 22 efficacy-evaluable patients with treatment duration ≥4 months, the ORR was 81.8%, and the CR rate was 36.4% (75). Combination therapy with AK117 and Aza displayed a manageable safety profile and significant efficacy as a first-line treatment in AML patients unable to undergo intensive chemotherapy, with a 55% complete clinical response rate (76).

### 5.3 Targeting CD47/SIRP *α* immunotherapy in multiple myeloma

Multiple myeloma (MM) has been considered to be incurable despite recent therapeutic advances. Preclinical studies targeting CD47/SIRPα signaling pathway related immunotherapy has been explored in MM patients. Study demonstrated that bortezomib-resistant multiple myeloma patient-derived xenograft is sensitive to anti-CD47 therapy (77). Blocking of CD47 using an anti-CD47 antibody induced immediate activation of macrophages showed phagocytosis and killing of MM cells in the 3D-tissue engineered bone marrow model (78). The tumor microenvironment plays a critical role in disease progression in MM. Single-cell atlas of the immune microenvironment reveals macrophage reprogramming and the potential dual macrophage-targeted strategy in MM by combining an anti-CD47 antibody and MIF (macrophage inhibitory factor) inhibition. The dual macrophage-targeted approach blocking both CD47 and MIF showed potent antitumor effects (79). Delivery of CD47-SIRPα checkpoint blocker by BCMA-directed universal chimeric antigen receptor T (UCAR-T) cells enhances antitumor efficacy in multiple myeloma in the xenograft model. Two possible mechanisms include the UCAR-T cells directly killed tumor cells and enhanced the phagocytosis of macrophages by secreting anti-CD47 nanobody hu404-hfc fusion (80).

A fully human CD38xCD47 bispecific antibody, ISB 1442, consists of two anti-CD38 arms targeting two distinct epitopes that preferentially drive binding to tumor cells and enable avidity induced blocking of proximal CD47 receptors on the same cell while preventing on-target off-tumor binding on healthy cells. Combined strategies for effective cancer immunotherapy with IBI188 have been considered as a potential treatment for MM patients (81). ISB 1442 is currently in a phase I clinical trial in R/R MM (NCT05427812) (82). In addition, a phase II multi-arm study of Magrolimab combinations in patients with R/R MM is under study (NCT04892446) (83). Another trial (NCT05139225) with the SIRPα/Fc fusion protein TTI-622 in combination with daratumumab hyaluronidase-fihj in patients with MM is ongoing. More extensive studies are needed regarding the efficacy of these agents targeting CD47/SIRPα signaling pathway in patients with MM.

## 6 Challenges of CD47/SIRPα immune checkpoint in cancer immunotherapy

Despite the promising outcomes observed in preclinical and clinical trials, the journey of developing CD47 mAbs has been riddled with challenges, notably centered around therapeutic efficacy and safety concerns. The persistent obstacles of toxicities pose ongoing challenges, with hematotoxicity, particularly anemia, and off-target myelosuppression emerging as predominant AEs associated with CD47 inhibitors. The widespread expression of CD47 on normal cells, particularly on red blood cells (RBCs) and platelets, creates a scenario where CD47 antagonists can directly target these cells or induce their recognition by natural killer (NK) cells and macrophages through Fc-mediated ADCC or complement dependent cytotoxicity (CDC) (84). The resultant hemolysis, a known consequence of CD47 mAbs, has led to the implementation of RBC pruning strategies in AML trials (2). This innovative approach involves a Magrolimab priming dose-escalation regimen, wherein the dosage is gradually increased from 1 to 30 mg/kg administered intravenously weekly (85, 86). This is followed by a maintenance dose of 30 mg/kg every 2 weeks in subsequent cycles beyond the third, aiming to alleviate the on-target anemia effects (63). The meticulous orchestration of dosing regimens reflects the dedication to mitigating adverse impacts, emphasizing the ongoing commitment to refining the therapeutic profile of CD47 antagonists in the pursuit of safer and more effective treatments. Several CD47-targeted clinical trials have been terminated, due to the safety concerns regarding the RBC binding activity and other adverse effects (87) (**Table 7**). Especially, the development of Magrolimab was interrupted in patients with AML and MDS and the related trials were discontinued for futility, non-efficacy, and increased mortality related to infections and respiratory problems. Overall, challenges remain to be overcome for the CD47/SIRPα related checkpoint inhibition immunotherapy, especially in AML (88).

**Table 7:** Targeting CD47 related clinical trials have been terminated.

Agent	MOA	Fc isotype	RBC binding	NCT#	Phase	Indications	Current status
Hu5F9	mAb targeting CD47	IgG4	Yes	NCT03248479	I	Hematological malignancies	Terminated
				NCT05835011	II	MDS	Terminated
				NCT04313881	III	MDS	Terminated
				NCT05079230	III	AML	Terminated
				NCT04778397	III	TP53 mutant untreated AML	Terminated
TTI-621	WT SIRPα-Fc fusion protein	IgG1	No	NCT02663518	I	Hematologic malignancies, solid tumor	Terminated
				NCT02890368	I	R/R solid tumors and mycosis fungoides	Terminated
				NCT04996004	II	Leiomyosarcoma	Terminated
TTI-622	WT SIRPα-Fc fusion protein	IgG4	No	NCT05261490	I, II	Ovarian cancer	Terminated
CC-90002	mAb targeting CD47	IgG4	Yes	NCT02641002	I	AML and high-risk MDS	Terminated
SHR1603	mAb targeting CD47	IgG4	Yes	N/A	N/A	Hematologic malignancies, solid tumor	Terminated
AO-176	mAb targeting CD47	IgG2	Yes	NCT03834948	I/II	Solid tumor	Terminated
				NCT04445701	I/II	R/R MM	Terminated
IBI188	mAb targeting CD47	IgG4	Yes	NCT04861948	I	Solid tumors	Terminated
TJC4	mAb targeting CD47	IgG4	Minimal	NCT04895410	I	MM	Terminated
				NCT04912063	I	AML and MDS	Terminated
Gentulizumab	mAb targeting CD47	IgG1	Minimal	NCT05221385	I	Advanced solid tumor and NHL	Terminated

MOA, mechanism of action; RBC, red blood cell; WT, wild type; mAb, monoclonal antibody; AML, acute myeloid leukemia; MDS, myelodysplastic syndrome; R/R, refractory/relapsed; MM, multiple myeloma; NHL, non-Hodgkin lymphoma.

Beyond the immunological challenges associated with CD47 antagonists, the inhibition of SIRPα introduces potential concerns related to the nervous system (86). Given the elevated expression of SIRPα on cells within both the central and peripheral nervous systems, the impact of SIRP inhibition on neural tissues becomes a notable consideration. The inherent structural similarities among members of the SIRP family further raise the prospect of improper interactions when CD47, present on cells, reacts with other SIRP members (81).

The conserved sequences within the SIRP family highlight the need for a nuanced approach to avoid unintended consequences. Recent insights into human T cell activation and proliferation underscore the favorable regulatory role played by CD47 when binding to SIRPα, particularly in comparison to the beta or gamma variants. The potential inhibition of T-cell activity, a critical component of the immune response, underlines the importance of thorough investigation in future research. To address these challenges and enhance therapy effectiveness while minimizing toxicity, the development of innovative strategies such as SIRPα/Fc fusion protein antibodies and CD47-targeted BsAbs is being explored (89, 90).

These novel approaches aim to strike a balance between therapeutic impact and safety, offering promising avenues to navigate the complexities associated with CD47-SIRPα axis modulation (62). However, the administration of higher dosages or frequent treatments carries the risk of inflicting damage on healthy cells, leading to potential unfavorable treatment-related consequences. Moreover, the effective dosage of CD47 antagonists may require adjustments in scenarios where tumor cells express both SIRPα and thrombospondin-1 (TSP-1), both of which disrupt the CD47-SIRPα interaction. To adequately activate macrophages, it becomes imperative to engage Fc receptors. Consequently, the choice of the appropriate human Immunoglobulin G (IgG) subtype plays a pivotal role (48, 55). While human IgG1 stimulates macrophages more effectively, it also heightens the risk of immune cells targeting RBCs. In response to this challenge, most companies opt for the development of human IgG4-type antibodies (55, 91). This strategic selection aims to strike a balance between the desired immune activation and the potential off-target effects, emphasizing the nuanced considerations involved in CD47-targeted therapies.

## 7 The rational analysis of the recent failures in CD47 mAbs clinical trials

### 7.1 The limitations and potential strategies for improvement

The safety concerns and incidents during the development of CD47-targeted drugs highlight the challenges in bringing these therapies to clinical applications. Instances like the premature termination of the phase 1 clinical trial of Ti061 (CD47 mAb) in 2017 due to unexpected deaths (92)and the failure of CD47 mAb CC90002 in a phase I clinical trial (NCT02641002) because of severe hemagglutination emphasize the importance of safety considerations (69, 70).

The favorable phase 1b results of Magrolimab in combination with Aza in patients with MDS and AML sparked enthusiasm for CD47-targeted drug development. The use of a low priming dose to remove aged erythrocytes and induce compensatory hematopoiesis (17, 63), along with the subsequent maintenance dose, led to Magrolimab receiving a Breakthrough Therapy designation for the treatment of newly diagnosed MDS in 2020. However, a partial clinical hold was placed on studies evaluating the combination of 5F9 plus Aza in January 2022 due to an apparent imbalance in suspected unexpected severe adverse reactions (93). Although concerns were initially linked to the hematotoxicity of Magrolimab or the additive toxicity of Aza, the partial clinical hold was removed in April 2022 after a comprehensive safety data review (94). In July 2023, the phase 3 ENHANCE study of Magrolimab in HR-MDS was discontinued (19). In August 2023, a partial clinical hold was placed on the recruitment of new patients in U.S. studies evaluating Magrolimab to treat AML (95).

There are several potential reasons for the inadequate efficacy of Magrolimab in recent clinical trial failures for cancer immunotherapy. These include tumor microenvironment complexity, heterogeneity of cancer cells, adaptive resistance, insufficient drug delivery and penetration, myeloid cell dysfunction, immune system modulation, off-target effects and toxicity, and clinical trial design. Further research and analysis of clinical trial data are necessary to pinpoint the exact reasons for the inadequate efficacy observed in these trials. Understanding these factors will help refine future therapeutic strategies and improve the design of subsequent trials.

Despite these challenges, CD47-targeted drugs continue to be a focus of tumor immunotherapy. Various strategies, such as anti-SIRPα mAbs, SIRPα-fusion proteins, BsAbs, dual ICIs, dual CAR-T cells, and OC-CD47, are being explored to overcome current limitations. Additionally, TAX2, the first antagonist of the TSP-1/CD47 axis, has been engineered. TAX2 selectively targets tumor-overexpressed TSP-1, inhibiting tumor angiogenesis and activating the anti-tumor immune system without harming blood cells (96).

In summary, the development of CD47-targeted drugs involves addressing safety concerns, overcoming limitations, and exploring innovative strategies to enhance tumor immunotherapy. Ongoing research and clinical trials aim to optimize the therapeutic potential of CD47-targeted therapies for various hematological malignancies and solid tumors.

### 7.2 The opportunity for antibodies targeting CD47/SIRP *α* pathway

**Figure 4:**
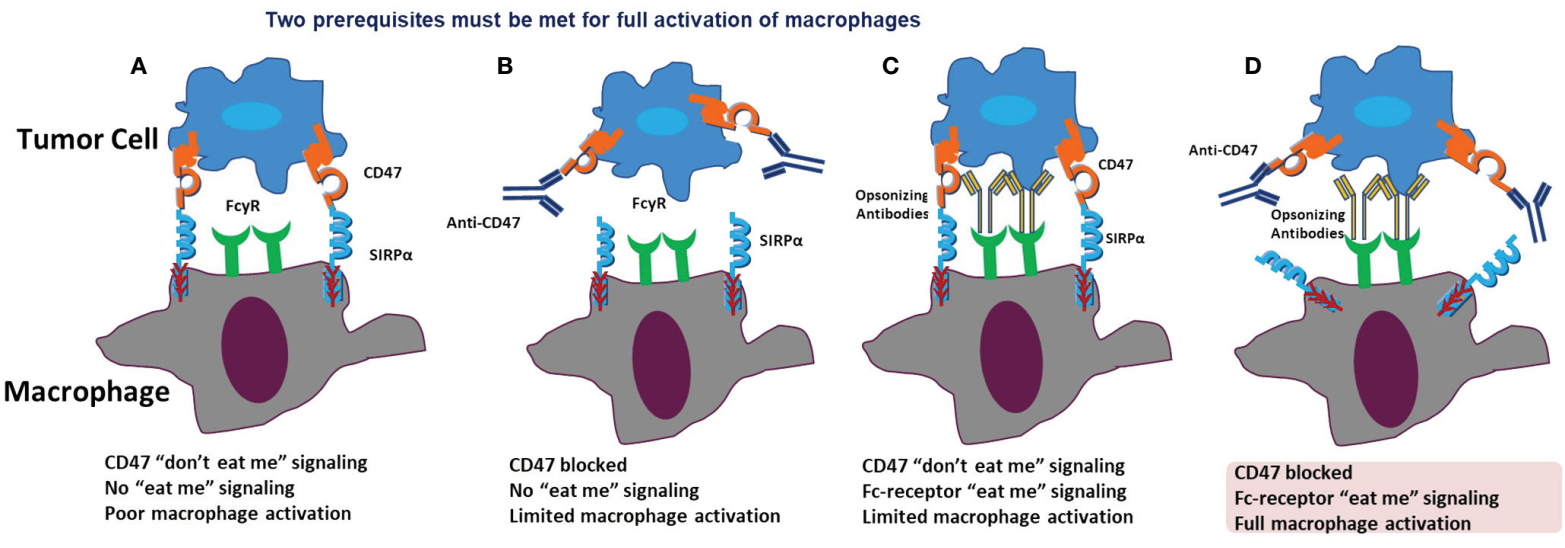
Prerequisites for macropage activation. In a normal situation, CD47 binds to SIRPα, acting as the “don’t eat me” signal, and no “eat me” signal is present. Therefore, there is no or poor macrophage activation **(A)**. When only CD47 is blocked, the “don’t eat me” signal is inhibited. However, no “eat me” signal is activated, resulting in only limited macrophage activation **(B)**. When the CD47 “don’t eat me” signal is present, and only the Fc-receptor “eat me” signal is activated, there is still only limited macrophage activation **(C)**. When both CD47 is blocked and the Fc-receptor “eat me” signal is activated, full macrophage activation occurs **(D)**.

In our exploration of drug actions targeting the CD47/SIRPα signaling pathway for tumor immunotherapy, we propose a hypothesis on macrophage activation. Full macrophage activation for effective tumor cell elimination hinges on two prerequisites: blocking the CD47 ‘don’t eat me’ signal and activating the Fc-receptor ‘eat-me’ signal. In the typical tumor cell environment, where tumor cells express CD47 and macrophages express SIRPα, there is an absence of the ‘eat-me’ signal. Limited macrophage activation occurs when either the CD47 ‘don’t eat me’ signal is blocked or the Fc-receptor ‘eat-me’ signal is activated. Only when both signals are manipulated—blocking CD47 and activating the Fc-receptor—do macrophages achieve full activation for effective tumor killing (**Figure 4**).

Despite the discontinuation of the phase 3 ENHANCE study of Magrolimab in MDS, there is still a glimmer of hope as several other key clinical trials, including AML, are progressing on track. CD47 is highly expressed on tumor cells and relatively low in peripheral blood cells. The receptor occupancy (RO) of CD47-targeting drugs (antibodies or SIRPα Fc recombinant protein) is predominantly detected by peripheral blood cells. The RO value does not directly correspond to the therapeutic effect of the drug but rather has a direct correlation with the “antigen sink” effect. Therefore, using peripheral blood to detect the RO of CD47-targeting drugs in clinical practice lacks therapeutic reference value.

Targeting the CD47/SIRPα signaling pathway continues to hold significant promise in solid tumor immunotherapy. When the selected mAb exhibits a moderate affinity with CD47 (in the nanomolar range) and does not excessively bind to red blood cells, it remains promising to determine an appropriate dosage for combination therapy with other drugs, such as IgG1 monoclonal antibodies like rituximab and Herceptin.

In contrast to antibodies, recombinant proteins targeting CD47, like IMM01, present a greater opportunity. IMM01, a recombinant human SIRPα IgG1 fusion protein, comprises SIRPα domain 1 and humanized IgG1 Fc segments. IMM01 demonstrates multifunctionality by blocking the CD47-SIRPα signaling pathway to avoid the “don’t eat me” signal. Additionally, it activates the “eat me” signal through Fc binding to the Fcγ receptors on the macrophage membrane, leading to the activation of macrophages. This activation, in turn, facilitates the presentation of tumor antigens to T cells, inducing tumor-specific T cell anti-tumor immunity (9, 97).

IMM01 presents several potential advantages over other agents in the realm of CD47-targeted immunotherapy. Firstly, it avoids the “antigen sink” risk, as it does not bind to red blood cells, and its target affinity is moderate. In comparison to Magrolimab, which exhibits a binding affinity range of 2–14.3 pM, IMM01 has a maximum binding affinity of 3 nM. The average receptor occupancy of peripheral PBMC cells is less than 20%, resulting in no significant ‘antigen sink’ effect. Secondly, owing to the unique molecular design of IgG1-Fc, the clinical recommended phase II dose (RP2D) of IMM01 is considerably lower than that of Magrolimab (IMM01: 2mg/kg; Magrolimab: 1mg/kg+30mg/kg), demonstrating good clinical safety and efficacy. Preliminary clinical trial results indicate a significant reduction in the incidence of grade 3 anemia with no cases of hemolytic anemia. Notably, IMM01 does not require priming dose administration. The stimulation of macrophages via IMM01 provides a scientific rationale for combining it with PD-1 monoclonal antibodies. This combination leads to the production of certain chemokines (CXCL9/CXCL10), driving T lymphocytes into tumor tissue and transforming ‘cold tumors’ into ‘hot tumors.’ Simultaneously, macrophages present tumor antigens processed by phagocytosis to T cells. Based on preliminary clinical trial data in patients with classic Hodgkin’s lymphoma relapsed after PD-1 antibody treatment, a synergistic effect with IMM01 has been observed, demonstrating an improved overall response rate from 17% to over 60% (unpublished data).

Given the current available information, predicting the outcome of Magrolimab+Aza therapy in AML appears challenging. The development of anti-CD47 antibody drugs faces inevitable safety issues, particularly when the binding ability of the antibody to red blood cells is too strong, causing an “antigen sink” effect where antibodies are predominantly taken by normal cells, leaving a minimal effective amount for tumor tissue. Higher doses may result in increased blood toxicity, including anemia and neutropenia. The root of the problem is believed to lie in the antibody itself, as IgG4 antibodies for the Fc portion do not encounter such issues.

## 8 Future perspectives

In the foreseeable future, CD47/SIRPα immunotherapy holds the potential to stand as a promising option, on par with PD-1/PD-L1. Enhancing the efficacy and safety of CD47 antagonists requires strategic development, and we propose several potential tactics for future exploration, including prime and maintenance dosing strategy, structure modification, dual blockade strategies (48, 98), BsAbs and fusion proteins (89), small-molecule inhibitors, novel drug delivery systems (49, 98–100), SIRP-1 and SIRP-2 applications (98), cis and trans interactions (49, 98, 99), and tumor selectivity enhancement (6) (**Table 8**). Furthermore, a combination of blockade of the CD47/SIRPα axis and secondary targets in the tumor microenvironment may also improve the clinical efficacy of current immunotherapeutic approaches (95).

**Table 8:** Potential tactics for future exploration of CD47-targeted therapeutic agents.

Potential tactics	Possible exploration	References or examples
Prime and maintenance dosing strategy	Priming and maintenance dosing exploration; Strategy exploration; Further validation of safety and efficacy.	5F9 and IBI-188
Drug structure modification	Evaluating the safety and efficacy of CD47 monoclonal antibodies with novel structures, such as AO-176, TJ011133, SRF231, and AK117	AO-176, TJ011133, SRF231, and AK117
Dual blockade strategies	Investigating the dual blockade targeting the CD47/SIRPα axis with fusion proteins like IMM0306 and NI-1701; Coupling with various antibodies such as CD20 and CD19	(48, 98)
Bispecific antibodies and fusion proteins	Developing CD47-targeted bispecific antibodies and SIRPα/Fc fusion proteins	(89)
Small-molecule inhibitors	Developing small-molecule inhibitor antibodies with good efficacy and safety	RRX001 and QPCTL
Novel drug delivery systems:	Deleloping innovative drug delivery systems, such as CD47 nanobodies, plasmid vectors, and CD47/SIRP blocking peptides	(49, 99`–101)
Sirp-1 and sirp-2 applications	Exploring the potential applications of SIRP-1 and SIRP-2 in cancer immunotherapy by disrupting the interaction between SIRPγ and CD47 while maintaining SIRP’s role in T cell activation	(102).
Cis and trans interactions	Developing dual nature of SIRPα interactions with CD47 in both cis and trans behaviors	(103–105)
Enhanced tumor selectivity	Increasing the tumor selectivity of CD47 antagonists, such as developing a pH-dependent CD47 antibody targeting tumor cells	(6).

## 9 Conclusion

In summary, the intricate dance of the CD47-SIRPα axis shields malignancies from immune scrutiny by issuing ‘don’t eat me’ signals that dampen macrophage phagocytosis. Overcoming these evasive tactics demands ongoing exploration of innovative cancer immunotherapeutic strategies targeting this axis, fueled by dedicated research and clinical trials. The current constraints of available treatments underline the pressing need for advancements. Furthermore, the specificity of CD47-targeted BsAbs limited to certain tumor types underscores the critical importance of precise malignancy identification and classification. As CD47 BsAbs progress through phases I and II clinical trials, the quest for the optimal dosage of dual antibodies becomes paramount.

Navigating this dynamic landscape calls for a profound understanding of the immune system, coupled with sophisticated designs for CD47 antagonists. This sets the stage for the emergence of new anti-CD47 drugs characterized by enhanced efficacy and safety. The notable progress achieved by innovative agents like IMM01 serves as a beacon of hope for patients facing unmet clinical needs. The future unfolds with the promise of transformative breakthroughs in cancer therapeutics, propelled by our ongoing exploration of the intricate interplay between CD47 and SIRPα.

## Author contributions

CJ: Data curation, Investigation, Writing – original draft, Writing – review & editing. HS: Data curation, Investigation, Writing – original draft, Writing – review & editing. ZJ: Writing – review & editing. WT: Conceptualization, Writing – review & editing. SC: Conceptualization, Resources, Supervision, Writing – review & editing. JY: Conceptualization, Funding acquisition, Resources, Supervision, Writing – review & editing.
